# Disassembly and rewiring of a mature converging excitatory circuit following injury

**DOI:** 10.1016/j.celrep.2021.109463

**Published:** 2021-08-03

**Authors:** Luca Della Santina, Alfred K. Yu, Scott C. Harris, Manuel Soliño, Tonatiuh Garcia Ruiz, Jesse Most, Yien-Ming Kuo, Felice A. Dunn, Yvonne Ou

**Affiliations:** 1 Department of Ophthalmology, University of California, San Francisco, San Francisco, CA 94158, USA; 2 Bakar Computational Health Sciences Institute, University of California, San Francisco, San Francisco, CA 94158, USA; 3 Neuroscience Graduate Program, University of California, San Francisco, San Francisco, CA 94158, USA; 4 Lead contact

## Abstract

Specificity and timing of synapse disassembly in the CNS are essential to learning how individual circuits react to neurodegeneration of the postsynaptic neuron. In sensory systems such as the mammalian retina, synaptic connections of second-order neurons are known to remodel and reconnect in the face of sensory cell loss. Here we analyzed whether degenerating third-order neurons can remodel their local presynaptic connectivity. We injured adult retinal ganglion cells by transiently elevating intraocular pressure. We show that loss of presynaptic structures occurs before postsynaptic density proteins and accounts for impaired transmission from presynaptic neurons, despite no evidence of presynaptic cell loss, axon terminal shrinkage, or reduced functional input. Loss of synapses is biased among converging presynaptic neuron types, with preferential loss of the major excitatory cone-driven partner and increased connectivity with rod-driven presynaptic partners, demonstrating that this adult neural circuit is capable of structural plasticity while undergoing neurodegeneration.

## In brief

Della Santina et al. injure a converging excitatory circuit in the adult retina by intraocular pressure elevation. Postsynaptic retinal ganglion cells disconnect from presynaptic bipolar cells with stereotyped bias against their major partner and rewire with developmental presynaptic partners, underscoring the potential of the adult CNS to adopt developmental patterns.

## Graphical abstract

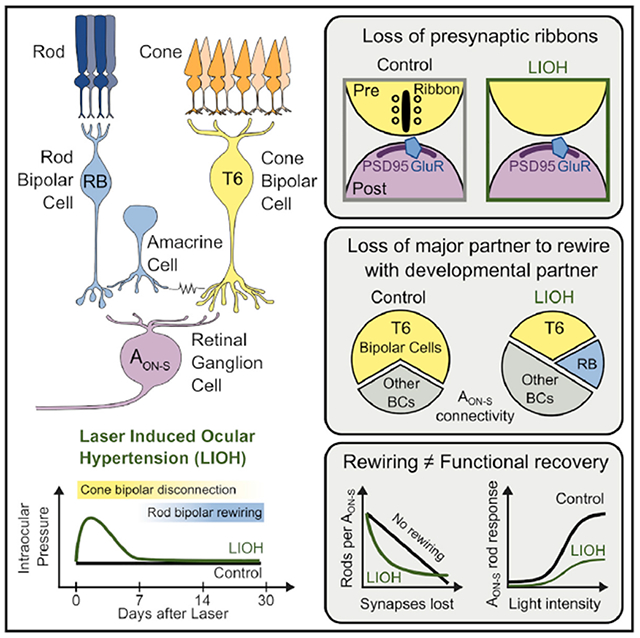

## INTRODUCTION

Circuit remodeling is a conserved strategy used to refine developing neural circuits across the nervous system. Axon, dendrite, and synapse elimination during development sculpt neural circuit assembly of vertebrate and invertebrate nervous systems (Colman and Lichtman, 1993; Riccomagno and Kolodkin, 2015; Sanes and Lichtman, 1999). While developing circuits typically eliminate aberrant or extraneous synaptic connections during a critical period of refinement, miswired and/or functional connections can also form between resilient or regenerating neurons after injury (Beier et al., 2017, 2018; Haverkamp et al., 2006; Shen et al., 2020; Yoshimatsu et al., 2016). Indeed, the effects of loss of neurotransmission and deafferentation of neural circuits have been studied in development but remain relatively unexplored in adult tissue. Here, we use a well-characterized circuit in the retina to investigate the extent to which presynaptic inputs remodel with degenerating postsynaptic neurons, quantifying the rewiring of presynaptic bipolar cells (BCs) and alterations of their synapses when their postsynaptic neurons, retinal ganglion cells (RGCs), are degenerating.

The effect of neurotransmission loss and presynaptic neuron ablation on connectivity has been studied in development but less so in adulthood (Johnson et al., 2017; Kerschensteiner et al., 2009; Okawa et al., 2014; Shen et al., 2020; Tien et al., 2017). When presynaptic neurons are injured or ablated, the postsynaptic neuron may reconnect with other healthy presynaptic partners. In the outer retina, loss of photoreceptors leads to structural and synaptic plasticity and dendritic remodeling in second-order BCs (Care et al., 2019). After laser ablation of photoreceptors, deafferented rod BCs form new synapses with healthy rods outside of the lesion (Beier et al., 2017) and can form ectopic synapses with cones (Haverkamp et al., 2006). However, very little is known about inner retina remodeling, and specifically rewiring, in situations where RGCs, third-order neurons, are damaged. Injury to the RGC is the hallmark of glaucoma, with synapse loss and dendritic shrinkage recognized as early events in RGC degeneration (Della Santina et al., 2013; Ou et al., 2016).

Glaucoma and optic nerve injury results in selective damage of specific RGC types (Della Santina and Ou, 2017; Della Santina et al., 2013; El-Danaf and Huberman, 2015; Ou et al., 2016; Tran et al., 2019). Across experimental models, alpha ON-sustained (A_ON-S_) RGCs emerged as one of the most resilient RGC types. For this reason, and the fact that its connectivity and light responses are well characterized, we focused our examination on A_ON-S_ RGCs. Here, we used a laser-induced ocular hypertension (LIOH) model in adult mice, which transiently elevates intraocular pressure (IOP), allowing us to assess the adult retina’s potential for remodeling and/or rewiring connectivity with presynaptic BCs after IOP returned to baseline. Morphological reconstructions and patch-clamp recordings demonstrated that presynaptic proteins were lost before postsynaptic proteins, the major presynaptic partner of the A_ON-S_ RGC was disconnected, and rewiring with developmental presynaptic partners occurred, although such rewiring was insufficient for functional recovery. These experiments suggest that the adult retina is capable of structural plasticity in the early phases of neurodegeneration, which is important for efforts in functional retinal integration aimed at vision restoration via cell-based therapies.

## RESULTS

**Figure 1.**
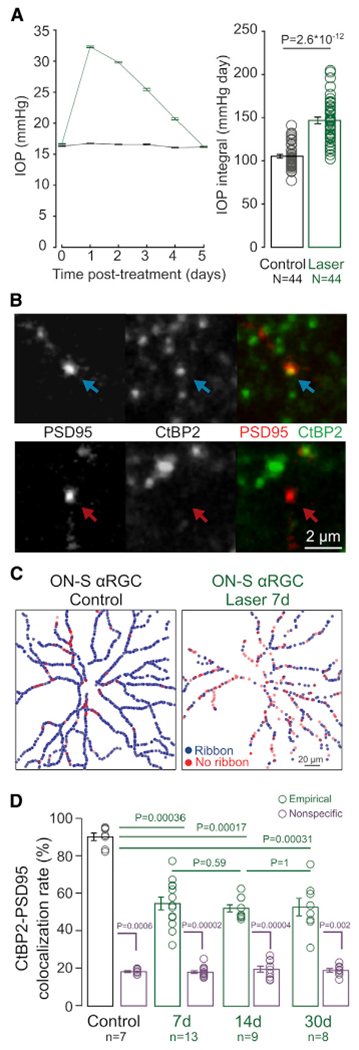
Disassembly of A_ON-S_ RGC synaptic sites after IOP elevation (A) IOP as a function of time (left panel) and average IOP integral (right panel). N = number of animals. (B) Example of a postsynaptic excitatory site (PSD95-YFP) apposed to (top row) or missing (bottom row) a presynaptic ribbon (CtBP2). Scale bar: 2 μm. (C) Example colocalization maps between postsynaptic PSD95 and presynaptic CtBP2 in A_ON-S_ RGC from control (left panel) and LIOH (right panel) retinas. (D) Colocalization rates between PSD95 and CtBP2 in control retinas and 7, 14, and 30 days after IOP elevation (green), compared to nonspecific colocalization obtained by 90 degree rotation of the RBC channel (purple). Plots: mean ± SEM. Circles: individual values. p values: rank-sum comparisons. n = number of A_ON-S_ RGCs, from N = 7 animals per group.

To selectively injure RGCs, we used the laser-induced ocular hypertension model (Fu and Sretavan, 2010; Ou et al., 2016). This procedure results in significant transient IOP elevation in the lasered eyes (Figure 1A, green) as compared to contralateral untreated eyes (Figure 1A, black). Peak IOP occurred within the first 24 h from induction (Figure S1C) and returned to baseline 5 days after treatment. We focused our analyses on three time points: 7, 14, and 30 days after laser, allowing us to decouple the observed alterations in the circuit from the causative injury. This manipulation induces selective death of RGCs without discernibly affecting presynaptic neuron layers (Figure S1A, ONL and INL in Figure S1B) or the thickness of the synaptic layer between postsynaptic RGCs and presynaptic BCs (IPL, in Figure S1B). At the age used for these experiments, retinal layer thickness is comparable to younger mice (P90 versus P30, Figures S1A and S1B; STAR Methods).

Previous work provides evidence that both presynaptic ribbons found in BC axon terminals and postsynaptic density proteins on RGC dendrites are lost after transient IOP elevation (Ou et al., 2016). However, the timing of synapse disassembly is not known. We focused our analysis on a specific type of RGC, the A_ON-S_ RGCs because they are conserved across some species (Peichl, 1991), easy to identify due to their large cell body and characteristic stratification, and, crucially, its connectivity pattern with presynaptic BCs is known from early postnatal ages to adulthood (Morgan et al., 2011; Schwartz et al., 2012). In mature retina, A_ON-S_ RGCs receive their primary excitatory inputs from type 6 ON cone BCs (T6 BCs; major partner). First, we identified in control experiments the presence of intact excitatory ribbon synapses onto this neuron by biolistically transfecting PSD95 on individual A_ON-S_ RGCs, immunolabeling ribbons in the same tissue (with marker CtBP2), and then performing colocalization analysis to detect PSD95 apposed to a ribbon (Figure 1B, top row) versus PSD95 not apposed to a presynaptic ribbon (Figure 1B, bottom row). Following this approach, we created colocalization maps for every excitatory postsynaptic site on the entire dendritic arbor of each RGC (Figure 1C). The colocalization rate between these two synaptic proteins for A_ON-S_ RGCs is above 80%, and relatively constant in mature RGCs. However, as early as 7 days after laser treatment we observed a significant drop of PSD95 apposed to ribbons, which remains constant at least until 30 days after treatment. Notably, as reported in Figure S1D, the average volume of PSD95 on A_ON-S_ cells transiently increased at 7 days (0.43 ± 0.07 versus 2.04 ± 0.31 μm^3^, p = 0.006), while the average ribbon volume decreased significantly 30 days after IOP elevation (0.49 ± 0.04 versus 0.69 ± 0.02 μm^3^, p = 0.0002). At all time points, the empirically observed colocalization rate was significantly higher than nonspecific colocalization obtained by rotating the CtBP2 channel (Figure 1D). In addition, colocalization rate of PSD95 with the glutamate receptors GluR 2/3 (Figure S1E) is similar in control and laser A_ON-S_ RGCs, indicating that transfected PSD95-YFP is closely associated with glutamate receptor sites on A_ON-S_ RGCs irrespective of its colocalization status with presynaptic ribbons. These data suggest that IOP elevation significantly reduced the fraction of PSD95 apposed to presynaptic ribbons, indicating that loss of presynaptic proteins precedes that of postsynaptic structures for excitatory synapses.

**Figure 2.**
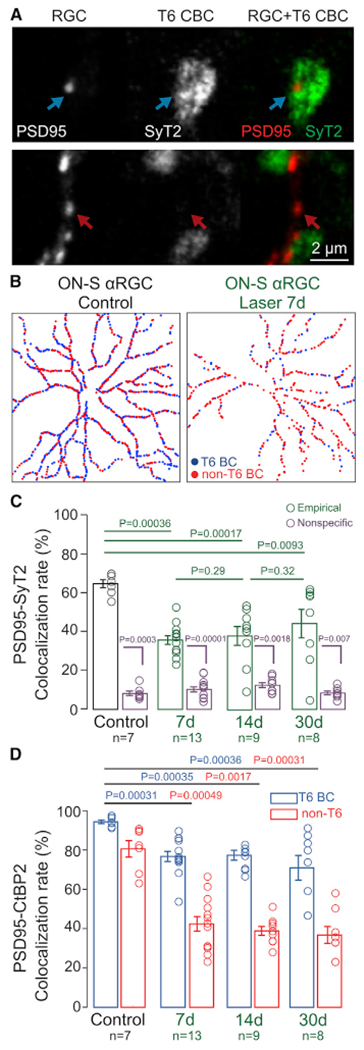
Biased disconnection of A_ON-S_ RGCs from presynaptic BC types (A) Top row: example of a postsynaptic excitatory site (PSD95-YFP) apposed to a presynaptic axon from T6 BCs (labeled by SyT2, top row) or to unlabeled (not T6) BC axon (bottom row). Scale bar: 2 μm. (B) Example connectivity maps between postsynaptic PSD95 and presynaptic T6 BCs in A_ON-S_ RGC from control (left panel) and LIOH (right panel) retinas. (C) Colocalization rates between PSD95 and T6 BCs in control retinas and 7, 14, and 30 days after IOP elevation (green), compared to nonspecific colocalization obtained by 90 degree rotation of the T6 BC channel (purple). (D) Rate of PSD95-CtBP2 colocalization for synapses apposed to T6 BCs (blue) and non-T6 BCs (red). Bar plots: mean ± SEM. Circles: individual values. p values: rank-sum comparisons. n = number of A_ON-S_ RGCs, from N = 7 animals per group.

After IOP elevation, synaptic contacts diminished between A_ON-S_ RGCs and their major excitatory presynaptic partner, T6 BCs. However, the complement of synaptic proteins in T6 BC synaptic terminals was not discernibly reduced. By biolistically labeling PSD95 on individual A_ON-S_ RGCs and immunolabeling T6 BC terminals with Synaptotagmin-2 antibody (Wässle et al., 2009), we differentiated PSD95 apposed to T6 BCs (Figure 2A, top row) from PSD95 not apposed to T6 BCs (i.e., apposed to other unlabeled BC types; Figure 2A, bottom row). This colocalization analysis was performed for every PSD95 on the dendrites of individual A_ON-S_ RGCs, providing a complete description of the connectivity pattern between these two neuron types (Figure 2B). In control adult retinas, the connectivity pattern of converging circuits was highly stereotyped for A_ON-S_ RGCs, with T6 BCs accounting for 60%–70% of all the excitatory synapses and, thus, providing the main excitatory drive to postsynaptic neurons (Schwartz et al., 2012). As soon as 7 days after IOP elevation, however, the residual connectivity with T6 BCs was significantly lower than control (35% ± 2% versus 64% ± 2%, p = 0.0004), demonstrating that A_ON-S_ RGCs lose a significant amount of input from their major partner (Figure 2C). The observed loss of connectivity with T6 BCs occurs in an environment where there is no significant loss of T6 BC axon terminal volume (Figure S2A) or coverage of retinal surface (Figure S2B). Similarly, there is no loss of ribbons within T6 BC axons (Figures S2C and S2D), indicating that disconnection from A_ON-S_ RGCs is highly specific and does not reflect a global loss of ribbons in these presynaptic axons.

**Figure 3.**
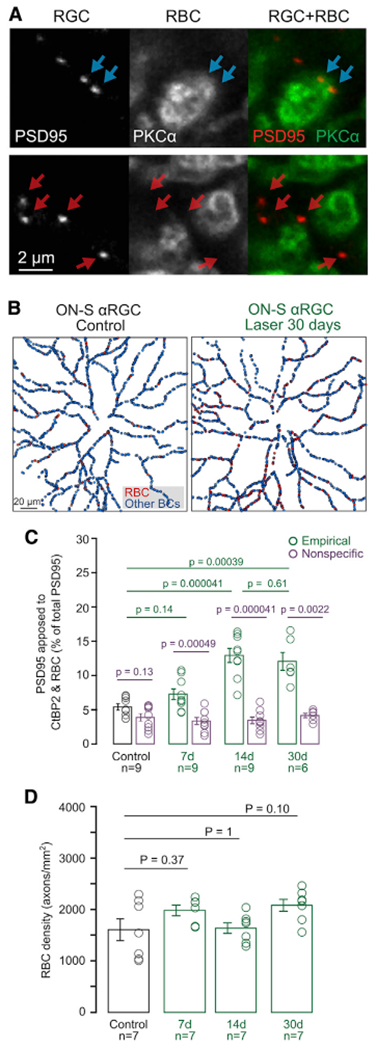
A_ON-S_ RGCs rewire with developmental partners, RBCs, but not because of increased RBC volume or ribbon density (A) Example of postsynaptic excitatory sites (PSD95-YFP) apposed to RBC axon terminals (top row) or to unlabeled (not-RBC) BCs (bottom row). Scale bar: 2 μm. (B) Example connectivity maps between postsynaptic PSD95 and presynaptic RBCs in A_ON-S_ RGC from control (left panel) and LIOH (right panel) retinas. (C) Fraction of PSD95 colocalized with RBCs in experimental data (green), compared to nonspecific colocalization level obtained by 90 degree rotation of the RBC channel (purple). Bar plots: mean ± SEM. Circles: individual RGC values. Rank-sum comparisons between groups indicated by lines. n = number of A_ON-S_ RGCs analyzed, from N = 7 animals per group. (D) RBC density measured by count of PKC-labeled axon stalks. n = number of retinas, from N = 7 animals per group.

Given the loss of input from T6 BCs, we wondered whether connectivity increased between A_ON-S_ RGCs and other presynaptic partners. Converging circuits usually attain their mature connectivity during development following an initial state of non-selective connectivity with all presynaptic partners stratifying in the same synaptic sublamina (Riccomagno and Kolodkin, 2015). Specifically, during development A_ON-S_ RGCs, which initially establish equal connectivity between T6, T7, and rod BCs (RBCs), progressively increase T6 connectivity at the expense of pruning RBC synapses (Morgan et al., 2011). We explored the possibility that during degeneration the connectivity pattern recapitulates the developmental program by labeling RBCs, mapping PSD95 apposed to their axon terminals (Figure 3A), and creating RBC-RGC connectivity maps (Figure 3B). First, we observed that even in control retinas there is a small percentage of direct connectivity with rod BCs (Figure 3C, black), although this is likely due to proximity of RBC axon terminals to A_ON-S_ dendrites in the ON sublamina of the IPL as seen by the nonspecific overlap calculated by rotating the RBC channel (Figure 3C, purple). A_ON-E_ RGCs progressively increase direct connectivity with RBCs, with a significant difference from both control and nonspecific overlap starting 14 days after IOP elevation. This process occurs in an environment where presynaptic RBC density remains constant (Figure 3D) along with their axon size, retinal surface coverage, and presynaptic ribbon density (Figure S3). Together, these findings document restorative connections between RBCs and A_ON-S_ RGCs, highlighting that this mature converging circuit undergoes stereotypic rearrangement of its connectivity during the early phase of degeneration.

**Figure 4.**
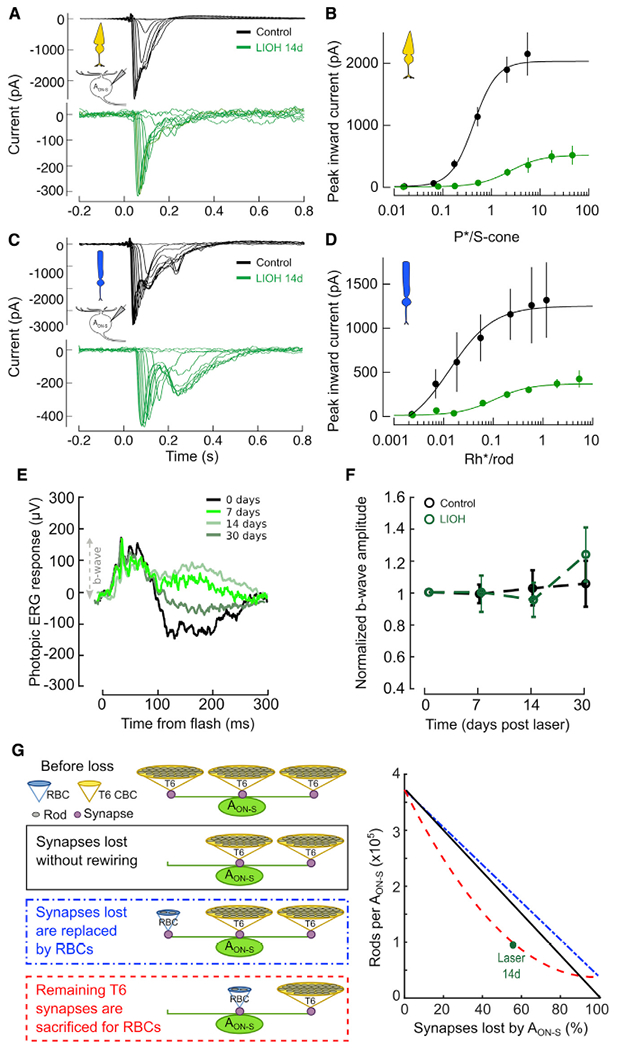
Light response of A_ON-S_ RGCs, but not of presynaptic BCs, is impaired (A and B) Cone-driven light responses in A_ON-S_ RGCs in response to 10 ms UV flashes of increasing light intensities on top of a blue rod-saturating background. Left panel: individual responses from representative RGCs. Right panel: peak inward current (mean ± SEM) as a function of isomerizations per s cone (P*/s-cone). solid lines: best-fit Hill equation (see STAR Methods). Excitatory currents were isolated by voltage clamping at −60 mV. n = recorded cells (7 control, 14 LIOH), from N = 10 animals. See also Table S2. (C and D) Rod-driven light responses in A_ON-S_ RGCs in response to 10 ms blue flashes of increasing light intensities in darkness. Left panel: individual responses from representative RGCs. Right panel: inward peak inward current (mean ± SEM) as a function of isomerizations per rod (Rh*/ rod). Solid lines: best-fit Hill equation (see STAR Methods). Excitatory currents were isolated by voltage clamping at −60 mV. n = recorded cells (7 control, 14 LIOH), from N = 10 animals. See also Table S2. (E) Left panel: example longitudinal traces of photopic ERG at different time points from LIOH. N = 6 animals per group. (F) Photopic b-wave amplitude relative to baseline as a function of time from LIOH. N = 6 animals. (G) Rod convergence onto A_ON-S_ RGCs via T6 CBCs and direct connectivity with RBCs as a function of BC-RGC synapse loss estimated in three case scenarios: after synapse loss without rewiring (black line); after synapses lost with T6 CBCs replaced by RBCs (blue line); after RBCs are rewired at the expense of remaining T6 BCs (red line). Green circle: estimated rod convergence from observed connectivity data.

We then explored the functionality of A_ON-S_ RGCs by recording light responses of individual cells in control retinas and in retinas 14 days after IOP elevation when rewiring with RBCs was observed. While A_ON-S_ RGCs from lasered retinas preserved their signature rod-mediated sustained response to a step of light impinging on their receptive field center, we observed a significant reduction in the magnitude of their excitatory input currents (Figure 4A–D). The reduced response of these cells occurred over their entire intensity-response function at both cone (Figure 4B) and rod (Figure 4D) light levels. To discriminate whether the observed reduction of light responses was due to impaired BC light response or reduced excitatory inputs onto A_ON-S_ RGCs, we recorded longitudinal electroretinograms (ERGs). Excitatory input current to the RGC measures all the ON BC inputs to the RGC, while ERG b-wave measures the current flow changes across the population of ON BCs, whether or not they provide input to the RGC. The photopic ERG response of lasered eyes at different times after IOP elevation was compared to the response of control contralateral eyes (Figure 4E), Quantification of the b-wave amplitude shows modest changes along time (Figure 4F, control versus laser, 7 days: 97% ± 5% versus 95% ± 10% p = 1; 14 days: 104% ± 9% versus 94% ± 9% p = 0.62; 30 days: 99% ± 14% versus 115% ± 17% p = 0.46), indicating that the specific functional defect of the cone pathway occurs in the connection between BCs and RGCs. To further explore whether the observed increase in direct connectivity between RBCs and A_ON-S_ RGCs occurs at the expense of cone BCs, we estimated the convergence from rods to A_ON-S_ RGCs (i.e., number of rods connected via the primary rod pathway per A_ON-S_ RGC) using connectivity data available in the literature as a function of percentage synapse loss by the A_ON-S_ RGCs (Figure 4G; see STAR Methods). We explored three case scenarios: (1) progressive loss of synapses without any RBC rewiring (Figure 4G, black trace), (2) addition of new synapses between A_ON-S_ RGCs and RBCs to exactly offsets all synapses lost between A_ON-S_ RGCs and T6 BCs (Figure 4G, blue trace), and (3) replacement of remaining A_ON-S_ RGC-T6 BC synapses with A_ON-S_ RGC-RBC synapses (Figure 4G, red trace). Empirical rewiring data observed 14 days after laser (Figure 4G, green circle) suggests that the rewiring strategy employed by A_ON-S_ RGCs is the last option, with RBCs gaining direct synaptic connectivity at the expense of remaining T6 BCs. This result also supports our finding that the light responses of A_ON-S_ RGCs fail to functionally recover after elevated IOP.

## DISCUSSION

Here, we demonstrate that upon IOP-induced injury, excitatory synapses of A_ON-S_ RGCs lose presynaptic ribbons prior to postsynaptic density proteins. Furthermore, synapse loss is biased, whereby A_ON-S_ RGCs lose connectivity with their major partner, T6 BCs. In addition, A_ON-S_ RGCs increase connectivity with RBCs, which are developmental but not adult presynaptic partners of this RGC type. However, this structural plasticity or rewiring occurs at the expense of convergence, thus preventing functional recovery as would be predicted. Our findings show that, upon RGC degeneration, adult presynaptic BCs disassemble presynaptic sites and rewire their connectivity, recapitulating developmental patterns during neurodegeneration. Future work is warranted to determine whether this finding is generalizable across circuits and to establish principles governing how damaged synaptic pathways might recover or form alternative circuits.

The finding that A_ON-S_ RGCs lose presynaptic ribbons before postsynaptic density proteins is surprising because the initial site of injury in glaucoma is the axon of RGCs. One possible interpretation of our findings is that, although the RGC degenerates and eventually dies, the presynaptic circuitry recognizes this injury and begins to disassemble synapses by removing ribbons. In mice lacking ribbons from development, A_ON-S_ RGCs have normal synapse density and respond to light stimuli, although less robustly with reduced frequency and contrast sensitivity (Okawa et al., 2019). This suggests that there may be a critical window prior to wholesale disassembly of both pre- and postsynaptic components of the synapse wherein RGC function is preserved. In contrast with our study, the microbead injection model, which results in lower IOP elevation for a longer period of time, shows a transient period in which presynaptic proteins increase while postsynaptic proteins go unchanged before synapse loss (Risner et al., 2018). This discrepancy could be explained by the different magnitude and chronicity of IOP elevation, time points examined, and analysis (normalized intensity measurement in the entire IPL done previously versus puncta quantification on individual RGCs done here). Furthermore, our study is limited to the specific protein markers examined. Additional studies using electron microscopy would further elucidate the order and extent of synapse disassembly at the BC-RGC synapse.

While homeostatic plasticity and the functional implications of biased loss of presynaptic input types for a neuron have been studied in development, our study fills a gap in understanding these phenomena in adult neurodegeneration. T6 BC ablation during development resulted in A_ON-S_ RGCs rewiring with new partners and increasing the number of synapses with existing partners, but retaining their functional hallmark (Tien et al., 2017). Here, we found that during adult neurodegeneration A_ON-S_ RGCs retain their rod-mediated sustained responses to light onset, preserving a recognizable physiological signature. However, rewiring does not compensate for loss of convergence, as evidenced by the lack of functional recovery. This may be due in part to the loss of convergence when the A_ON-S_ RGC rewires with rod BCs (Figure 4G), but it may also reflect intrinsic dysfunction and neurodegeneration of the RGC due to IOP-induced injury, as the number of excitatory synapses drops as early as 7 days after IOP elevation (Table S1). In contrast, models of primary neuron ablation show functional resilience in mature retina (Care et al., 2019), although it declines with age (Shen et al., 2020).

Loss of connectivity between A_ON-S_ RGCs and T6 BCs, together with rewiring with RBCs, may represent recapitulation of developmental preferences as a form of synaptic plasticity in response to injury. During synaptogenesis, A_ON-S_ RGCs establish connectivity with multiple partners and then selectively refine synapses to reach their mature connectivity pattern (Morgan et al., 2011). It is not known whether the direct rewiring with a developmental partner is an injury response intended to preserve synapses or a mechanism to slow RGC degeneration. Further work is needed to explore the extent to which the new synapses are functional and whether axons of other ON BC types also increase connectivity to A_ON-S_ RGCs. Loss of neurotransmission from BCs results in decreased density of pre- and postsynaptic proteins, but connectivity is not altered (Kerschensteiner et al., 2009; Okawa et al., 2014), while, when ON BCs were ablated during development, A_ON-S_ RGCs sprouted dendrites in the OFF sublamina forming aberrant functional connections with OFF BCs (Okawa et al., 2014). On the other hand, when the presynaptic neuron is ablated in the adult retina, there is cell-type and age-dependent rewiring of the postsynaptic neuron that occurs to restore homeostasis (Shen et al., 2020). In contrast to these tightly controlled manipulations, we find that the presynaptic ribbon density within T6 BC axons remains constant. Whether presynaptic BCs reassign ribbons to other postsynaptic partners is an intriguing possibility for adult homeostasis deserving of study.

Using this model of transient IOP elevation, we previously demonstrated that synapse loss precedes dendrite retraction and RGC loss (Ou et al., 2016), but it was not known whether the presynaptic circuitry was also perturbed. Our findings suggest that the current paradigm of glaucoma pathophysiology and vision restoration, focused on axonal injury at the optic nerve head and regeneration toward target neurons in the brain, should be expanded to include the presynaptic retinal circuitry. While experimental models of glaucoma vary in the chronicity and degree of IOP elevation and have limitations in their relevance to human glaucoma, including the transient IOP elevation model used here, several lines of evidence suggest that A_ON-S_ RGCs are resilient in the face of glaucomatous neurodegeneration (Della Santina et al., 2013; El-Danaf and Huberman, 2015; Ou et al., 2016; Tran et al., 2019). Our data suggest that they are not resilient because of preserved input from major presynaptic partners, but rather due to capacity to alter their connectivity pattern. Identifying cell-intrinsic factors underlying resilience versus vulnerability of specific RGC types, as well as recognizing windows in which structural plasticity is possible would be critical to develop novel neuroprotection treatments for glaucoma that are independent of lowering IOP, the mainstay and only treatment currently available.

## STAR★METHODS

### RESOURCE AVAILABILITY

#### Lead contact

Further information and requests for resources and reagents should be directed to and will be fulfilled by Luca Della Santina (luca.dellasantina@ucsf.edu).

#### Materials availability

The study did not generate new unique reagents.

#### Data and code availability

- All data reported in this paper will be shared by the lead contact upon request.
- All original code has been deposited at https://github.com/lucadellasantina and is publicly available as of the date of publication. DOIs are listed in the key resources table.
- Any additional information required to reanalyze the data reported in this paper is available from the lead contact upon request.

### EXPERIMENTAL MODEL AND SUBJECT DETAILS

#### Animals

Male and female CD-1 albino mice were purchased from Charles River Laboratories and housed in animal facilities at the University of California, San Francisco, following a standard diet and exposed to a daily light cycle of 12h dark-12h light. All experiments were conducted in female and male animals 2-3 months of age. All animal procedures were approved by the Institutional Animal Care and Use Committees at University of California, San Francisco.

### METHOD DETAILS

#### Laser-induced ocular hypertension

Mice were anesthetized with intraperitoneal injections of ketamine/xylazine and IOP measured for each eye using the Tonolab rebound tonometer (Colonial Medical Supply). The probe was triggered with a custom foot pedal to minimize movement of the instrument during IOP measurement. Three measurements (each an average of 6 readings) were taken. In order to cause transient obstruction of aqueous outflow, mice were placed under a surgical microscope and an endoprobe attached to a diode laser (532 nm; Lumenis) was used to photocoagulate the limbal and at least 3–6 episcleral vessels in the left eye (300 mW laser power, 0.5 s duration, 100 μm diameter spot size, total spots ~80-100). The translimbal laser treatment was performed over 330 degrees sparing the nasal aspect and the long posterior ciliary arteries. After the procedure, lubricant ophthalmic ointment was applied to the lasered eye. Each animal received only one laser photocoagulation treatment with the untreated contralateral eye serving as the control. IOP was monitored daily under isoflurane for 5-7 d and mice that demonstrated at least 30% increase in IOP followed by a decline to baseline were included in the study, whereas mice that developed an IOP > 50 mmHg were excluded. Mice with eyes exhibiting overt signs of corneal edema, hyphema, and inflammation were also excluded from the study.

#### Biolistic transfection

Mice were anesthetized by isoflurane and euthanized by cervical dislocation. Eyes were removed and placed in oxygenated mouse ACSF (pH 7.4), containing the following (in mm): 119 NaCl, 2.5 KCl, 1.3 MgCl_2_*6H_2_O, 2.5 CaCl_2_*2H2O, 1 NaHPO_4_, 11 glucose, 20 HEPES. Retinas were isolated from the eyecup under a dissection microscope and mounted onto nitrocellulose filter paper (Millipore). DNA-coated gold particles were prepared by coating 12.5 mg of 1.6 μm gold particles (Bio-Rad) with 20 μg of *CMV*:CFP and 7 μg of *CMV*:PSD95-YFP plasmids. A Helios gene gun (Bio-Rad) was used to biolistically deliver plasmid-coated gold particles to whole-mounted retinas. A suspension of DNA-coated gold particles in ethanol was precipitated onto the inner surface of Teflon tubing (Bio-Rad) and subsequently cut into short segments (12 mm long). Gold particles were propelled onto the tissue using helium gas at 40 psi. Retinas were then transferred to an oxygenated and humidified chamber and maintained for 26 h at 32°C, allowing fluorescent protein to be expressed sufficiently for subsequent imaging.

#### Ocular histology

Eyes were harvested at appropriate time points and fixed in half strength Karnovsky fixative (4% paraformaldehyde + 2.5% glutaraldehyde) in 0.1M PO_4_ buffer pH 7.4 for 48 hours, dehydrated in graded ethanol and embedded in Technovit 7100 Glycol Methacrylate (Electron Microscopy Sciences, Hatfield, PA). Embedded tissues were serially sectioned (2 μm) by passing through the optic nerve head and stained with Hematoxylin and Eosin (H&E). For each retina a section cut along the naso-temporal equator containing the optic nerve head was acquired by an upright Axiophot microscope (Zeiss, Germany) equipped with a Plan Neofluar 20X objective (NA 0.5). A blinded investigator quantified the thickness of the outer and inner nuclear layers and of the inner plexiform layer at ten locations equally spaced along the naso-temporal axis, and values for a given retina were expressed either as average number of nuclei (for nuclear layers) or average microns (for the inner plexiform layer).

#### Immunohistochemistry

For the biolistic transfection experiments, retinas were fixed in 4% PFA in ACSF for 20–30 min. Following an overnight block containing 5% normal donkey serum (Jackson ImmunoResearch), 2% BSA (Sigma), and 0.5% Triton X-100 (Millipore) in PBS, the retinas were incubated with the following primary antibodies for 4 nights: mouse monoclonal anti-CtBP2 antibody (1:1000; BD Biosciences), anti-synaptotagmin 2 (mouse 1:300, SyT2, ZIRC), anti-PKCa antibody (mouse 1:500; Novus Biologicals) or anti-GluR2/3 (rabbit 1:500, Millipore). After washing and incubating with the appropriate secondary antibodies (Alexa, Invitrogen, 1:1000; or Dylight, Jackson ImmunoResearch Laboratories, 1:1000, conjugated fluorophores) overnight at 4°C, retinas were washed again and mounted onto glass slides using Vectashield (Vector Laboratories). The same tissue was also used for bipolar axon terminal volume analysis (see supplemental methods).

#### Image acquisition and analysis

Confocal image stacks were acquired using an upright microscope (Leica SP8, Germany) with a 63X oil immersion microscope (NA 1.40), with a voxel size of 0.098 × 0.098 × 0.3 μm. A_ON-S_ RGCs were targeted and identified by their characteristic large somata and stratification level in the IPL (Ou et al., 2016). Image stacks were median filtered (kernel size 3×3 voxels) to remove thermal noise from the microscope detector and converted to 8-bit. For each acquired RGC, synaptic locations of PSD95-YFP puncta were identified using ObjectFinder following the procedure described in (Della Santina and Ou, 2017), Briefly, a digital skeleton of the entire dendritic arbor of the neuron was generated using Imaris (Bitplane, Zurich, CH). The skeleton of the cell was fitted to the cytosolic CFP signal to segment the full volume of the dendritic arbor. Within this volume, PSD95-YFP puncta were automatically recognized using Object-Finder’s 3D iterative thresholding algorithm. The obtained set of objects was visually inspected in 3D and validated by a trained observer. Colocalization analysis between PSD95 and CtBP2, as well as between PSD95 and SyT2 axons was manually performed using ObjectFinder by visual inspection of the local volume around each PSD95-YFP puncta and counts from at least two blinded observers were averaged for each individual neuron. Puncta volume and colocalization rates were measured in ObjectFinder and the average measurement was reported for each individual RGC.

For bipolar axon terminal quantification, image stacks were taken in similar locations in the central retina in each leaflet of the whole mount retina. Briefly, ImageJ was used to median filter the image stack, 3D Slicer to segment only the axon terminals, and Amira was used to create a binary mask via thresholding. ObjectFinder was used to identify CtBP2 puncta as described above and VolumeCut was used to analyze axon volume as well as volume occupancy of CtBP2 with the axon terminals. Area occupancy was determined by creating a z projection of the axon terminals and quantifying the area of the axon terminals in relation to the area of the field.

#### Patch clamp electrophysiology

Electrophysiology experiments were performed following previously described protocols (Care et al., 2019, 2020). Briefly, mice were dark-adapted overnight and subsequently euthanized by cervical dislocation and enucleated. Retinal dissections were performed in the dark with infrared converters and the tissue was placed on a glass cover slide with the ganglion cell layer facing up for patch-clamp recordings. During experiments the tissue was continuously perfused at 8-10 mL/min with bicarbonate-based Ames medium equilibrated with 95% O_2_/5% CO_2_ heated to 35°C. Putative A_ON-S_ ganglion cells were targeted for whole-cell recordings based on their large soma size (visualized under 950nm light) and sustained responses to 500ms steps of light in the cell-attached configuration. After cell type identification, recordings were switched to the voltage-clamp configuration using an internal solution of (in mM): 104.7 cesium methane sulfonate, 10 TEA Cl, 20 HEPES, 10 EGTA, 2 QX-314, 5 ATP, 0.5 GTP, adjusted to pH 7.3 with CsOH and 0.04% Lucifer Yellow dye. Ganglion cells were voltage-clamped at −60mV to isolate excitatory input currents. To check the quality of the recordings, the following parameters were measured for a subset of cells: capacitance (50.40 ± 14.01 pF, n = 12) and series resistance (9.81 ± 2.16 MOhm, n = 12; mean ± SEM). The average holding current was also compared between all control (−1757.0 ± 728.8 pA, n = 16) and LiOH (−1556.9 ± 476.1 pA, n = 16; mean ± SEM) cells with no significant difference between the populations (t test).

A 500μm spot of light was focused on the photoreceptor layer of the tissue using a blue (peak wavelength = 472nm) and/or UV (peak wavelength = 405nm) LED. For experiments probing rod-mediated responses, membrane currents were recorded in response to a series of 10ms flashes each doubling in intensity from the previous flash and presented from darkness using the blue LED. For experiments probing cone-mediated responses, cells were first adapted for several minutes to a rod-saturating light intensity using the blue LED (4000 Rh*/rod/sec), after which membrane currents were recorded in response to a series of 10ms flashes each doubling in intensity from the previous flash using the UV LED. Data were acquired using Symphony DAS. A 60Hz low-pass filter was applied to all membrane current traces. Experimenters were blinded as to which cells came from laser and control eyes.

Input current experimental data points were fitted with the following Hill function in Igor Pro:

$${\text{y}=\text{base}+(\text{max}-\text{base})/[1+(\text{x}}_{\text{half}}{/\text{x})}^{\text{rate}}\text{]}$$

Where x represents the flash intensity and y the recorded absolute current. Fit coefficients for cone-mediated light responses: Control base = 10.7 ± 4.4, max = 2033.2 ± 221, rate = 1.8429 ± 0.255, x_half_ = 0.43 ± 0.09); LIOH base = 6.92 ± 1.47, max = 519.41 ± 102, rate = 1.34 ± 0.23, x_half_ = 2.40 ± 1.1). Fit coefficients for rod-mediated light responses: Control: base = 189 ± 576, max = 1251 ± 344, rate = 0.99 ± 1.12, x_half_ = 0.013 ± 0.013 LIOH: base = 12.01 ± 7.15, max = 370.1 ± 48.1, rate = 1.22 ± 0.34, x_half_ = 0.105 ± 0.039).

#### Electroretinogram recording

Mice were anesthetized with intraperitoneal injections of ketamine/xylazine and positioned on the recording apparatus (Celeris, Diagnosys LLC, Lowell, MA). Pupils were dilated using 1% tropicamide and corneas kept moist through the recording with a thin layer of methylcellulose. The animal’s body temperature was constantly monitored and maintained at 37°C by a heating pad. Electroretinograms (ERGs) were recorded by electrodes making contact with the moist cornea.

A gold needle electrode was placed under the skin between shoulders to serve as both reference and ground. Responses were amplified differentially, band-pass filtered between 0.1 and 500 Hz, digitized at 10 kHz, and stored on a hard drive. Responses to flashes were averaged with an interstimulus interval of 10 s. Five to seven responses were averaged for each light intensity to eliminate electrical noise. Full field illumination of the eyes was achieved with the miniaturized Ganzfeld spheres integrated with the recording electrodes (Celeris Bright RGB stimulators, Diagnosys LLC). Brief (10ms) white flashes (97 photons/mm^2^/s) were delivered over a rod-saturating background (30 cd/m^2^), generating the typical flash ERG response (Ekesten et al., 1998-1999). The b-wave was measured from the negative a-wave peak to the peak of the large positive wave.

#### Rod pathway convergence model

In order to predict the effect on photoreceptor signal convergence of synaptic loss, we created a model in which the independent variables are the fraction of total excitatory synapses lost between cone bipolar cells and A_ON-S_ RGC (loss), and the fraction of total excitatory synapses on A_ON-S_ RGC gained by direct connection with rod bipolar cells (rewire). We then proceeded to calculate separately the synaptic convergence of cones and rods under these condition by the formula:

$${\text{C}}_{\text{Rod}\Rightarrow \text{AON}-\text{S}}(\text{loss},\text{rewire})={\text{C}}_{\text{Rod}\Rightarrow \text{RBC}}*{\text{C}}_{\text{RBC}\Rightarrow \text{All}}*\left[1-{\text{C}}_{\text{RBC}\Rightarrow \text{AON}-\text{S}}(\text{rewire})]*{\text{C}}_{\text{All}\Rightarrow \text{CBC}6}*{\text{C}}_{\text{CBC}6\Rightarrow \text{AON}-\text{S}}(\text{loss}\right)$$

Where C_Neuron1⇒Neuron2_ are the convergence factors for specific pairs of neurons. We used the following published convergence factors: Rod⇒RBC = 20 (Tsukamoto and Omi, 2017), RBC⇒All = 9 (Tsukamoto and Omi, 2013), All⇒CBC6 = 9.3 (Tsukamoto and Omi, 2013), CBC6⇒A_ON-S_ = 211 (Schwartz et al., 2012), Cone⇒CBC6 = 4 (Dunn and Wong, 2012).

### QUANTIFICATION AND STATISTICAL ANALYSIS

Unless otherwise stated, all measurements were reported as mean ± SEM. Pairwise comparisons were computed using the Wilcoxon–Mann–Whitney test. P values are reported as decimal numbers rounded to the second significant digit, with significance defined as p value < 0.01. Number of cells / animals are reported in each figure at the base of bar plots or in the figure legend for all other types of plots. Statistical analysis was computed using MATLAB R2020a (Mathworks, Natick, MA).

## Supplementary Material

1

## Figures and Tables

**Table:** KEY RESOURCES TABLE

Reagent or resource	Source	Identifier
**Antibodies**		
anti-CtBP2 mouse monoclonal antibody	BD Biosciences	Cat # 612044 RRID:AB_399431
anti-PKC alpha mouse monoclonal antibody	Novus Biologicals	Cat # NB600-201; RRID:AB_10003372
anti-PSD95 mouse monoclonal antibody	Neuromab	Cat# 73-028; RRID:AB_10698024
anti-Synaptotagmin-2 mouse monoclonal antibody	Zebrafish International Resource Center	Cat # ZDG-ATB-081002-25; RRID:AB_10013783
DyLight 405 AffiniPure Goat Anti-Mouse IgG, Fcγ subclass 1 specific	Jackson ImmunoResearch	Cat # 115-475-205; RRID:AB_2338799
Goat anti-Mouse IgG2a Cross-Adsorbed Secondary Antibody, Alexa Fluor 568	Invitrogen	Cat # A-21134; RRID:AB_2535773
anti-glutamate receptor 2&3 rabbit polyclonal antibody	Millipore	Cat # AB-1506; RRID:AB_90710
**Experimental models: Organisms/strains**		
CD-1 albino mice (Crl:CD1(ICR) Outbred)	Charles River Laboratories	RRID:IMSR_CRL:022
**Recombinant DNA**		
CMV:PSD95-YFP plasmid	Rachel Wong lab, University of Washington	N/A
CMV:tdTomato plasmid	Rachel Wong lab, University of Washington	N/A
**Software and algorithms**		
Imaris	Bitplane	RRID:SCR_007370
MATLAB	MathWorks	RRID:SCR_001622
VolumeCut	https://github.com/lucadellasantina/VolumeCut	https://doi.org/105281/zenodo.5048331
ImageJ	NIH	RRID:SCR_003070
ObjectFinder	https://github.com/lucadellasantina/ObjectFinder	https://doi.org/105281/zenodo.4767847
Symphony DAS	Mark Cafaro and Fred Rieke	https://github.com/Symphony-DAS/symphony-v1/wiki

## Highlights

- Upon injury, RGC dendrites lose presynaptic inputs before postsynaptic sites
- Synapse loss is biased among presynaptic types, skewed against major partners
- Reconnection with former developmental partners occurs in the adult circuit
- Rewiring occurs at the expense of convergence, preventing functional recovery

## References

[R1] Beier C, Hovhannisyan A, Weiser S, Kung J, Lee S, Lee DY, Huie P, Dalal R, Palanker D, and Sher A (2017). Deafferented Adult Rod Bipolar Cells Create New Synapses with Photoreceptors to Restore Vision. J. Neurosci. 37, 4635–4644. 28373392 10.1523/JNEUROSCI.2570-16.2017 PMC5413192

[R2] Beier C, Palanker D, and Sher A (2018). Stereotyped Synaptic Connectivity Is Restored during Circuit Repair in the Adult Mammalian Retina. Curr. Biol. 28, 1818–1824.e2. 29804805 10.1016/j.cub.2018.04.063 PMC6550309

[R3] Care RA, Kastner DB, De la Huerta I, Pan S, Khoche A, Della Santina L, Gamlin C, Santo Tomas C, Ngo J, Chen A, (2019). Partial Cone Loss Triggers Synapse-Specific Remodeling and Spatial Receptive Field Rearrangements in a Mature Retinal Circuit. Cell Rep. 27, 2171–2183.e5. 31091454 10.1016/j.celrep.2019.04.065 PMC6624172

[R4] Care RA, Anastassov IA, Kastner DB, Kuo Y-M, Della Santina L, and Dunn FA (2020). Mature Retina Compensates Functionally for Partial Loss of Rod Photoreceptors. Cell Rep. 31, 107730. 32521255 10.1016/j.celrep.2020.107730 PMC8049532

[R5] Colman H, and Lichtman JW (1993). Interactions between nerve and muscle: synapse elimination at the developing neuromuscular junction. Dev. Biol. 156, 1–10. 8449362 10.1006/dbio.1993.1054

[R6] Della Santina L, and Ou Y (2017). Who’s lost first? Susceptibility of retinal ganglion cell types in experimental glaucoma. Exp. Eye Res. 158, 43–50. 27319294 10.1016/j.exer.2016.06.006 PMC5161723

[R7] Della Santina L, Inman DM, Lupien CB, Horner PJ, and Wong ROL (2013). Differential progression of structural and functional alterations in distinct retinal ganglion cell types in a mouse model of glaucoma. J. Neurosci. 33, 17444–17457. 24174678 10.1523/JNEUROSCI.5461-12.2013 PMC3812509

[R8] Dunn FA, and Wong ROL (2012). Diverse strategies engaged in establishing stereotypic wiring patterns among neurons sharing a common input at the visual system’s first synapse. J. Neurosci. 32, 10306–10317. 22836264 10.1523/JNEUROSCI.1581-12.2012 PMC3435432

[R9] Ekesten B, Gouras P, and Moschos M (1998-1999). Cone properties of the light-adapted murine ERG. Doc. Ophthalmol. 97, 23–31. 10.1023/a:1001869212639 10710239

[R10] El-Danaf RN, and Huberman AD (2015). Characteristic patterns of dendritic remodeling in early-stage glaucoma: evidence from genetically identified retinal ganglion cell types. J. Neurosci. 35, 2329–2343. 25673829 10.1523/JNEUROSCI.1419-14.2015 PMC6605614

[R11] Fu CT, and Sretavan D (2010). Laser-induced ocular hypertension in albino CD-1 mice. Invest. Ophthalmol. Vis. Sci. 51, 980–990. 19815738 10.1167/iovs.09-4324 PMC2868470

[R12] Haverkamp S, Michalakis S, Claes E, Seeliger MW, Humphries P, Biel M, and Feigenspan A (2006). Synaptic plasticity in CNGA3(−/−) mice: cone bipolar cells react on the missing cone input and form ectopic synapses with rods. J. Neurosci. 26, 5248–5255. 16687517 10.1523/JNEUROSCI.4483-05.2006 PMC6674253

[R13] Johnson RE, Tien N-W, Shen N, Pearson JT, Soto F, and Kerschensteiner D (2017). Homeostatic plasticity shapes the visual system’s first synapse. Nat. Commun. 8, 1220. 29089553 10.1038/s41467-017-01332-7 PMC5663853

[R14] Kerschensteiner D, Morgan JL, Parker ED, Lewis RM, and Wong ROL (2009). Neurotransmission selectively regulates synapse formation in parallel circuits in vivo. Nature 460, 1016–1020. 19693082 10.1038/nature08236 PMC2746695

[R15] Morgan JL, Soto F, Wong ROL, and Kerschensteiner D (2011). Development of cell type-specific connectivity patterns of converging excitatory axons in the retina. Neuron 71, 1014–1021. 21943599 10.1016/j.neuron.2011.08.025 PMC3184549

[R16] Okawa H, Della Santina L, Schwartz GW, Rieke F, and Wong ROL (2014). Interplay of cell-autonomous and nonautonomous mechanisms tailors synaptic connectivity of converging axons in vivo. Neuron 82, 125–137. 24698272 10.1016/j.neuron.2014.02.016 PMC3990864

[R17] Okawa H, Yu W-Q, Matti U, Schwarz K, Odermatt B, Zhong H, Tsukamoto Y, Lagnado L, Rieke F, Schmitz F, and Wong ROL (2019). Dynamic assembly of ribbon synapses and circuit maintenance in a vertebrate sensory system. Nat. Commun. 10, 2167. 31092821 10.1038/s41467-019-10123-1 PMC6520400

[R18] Ou Y, Jo RE, Ullian EM, Wong ROL, and Della Santina L (2016). Selective Vulnerability of Specific Retinal Ganglion Cell Types and Synapses after Transient Ocular Hypertension. J. Neurosci. 36, 9240–9252. 27581463 10.1523/JNEUROSCI.0940-16.2016 PMC5005727

[R19] Peichl L (1991). Alpha ganglion cells in mammalian retinae: common properties, species differences, and some comments on other ganglion cells. Vis. Neurosci. 7, 155–169. 1931799 10.1017/s0952523800011020

[R20] Riccomagno MM, and Kolodkin AL (2015). Sculpting neural circuits by axon and dendrite pruning. Annu. Rev. Cell Dev. Biol. 31, 779–805. 26436703 10.1146/annurev-cellbio-100913-013038 PMC4668927

[R21] Risner ML, Pasini S, Cooper ML, Lambert WS, and Calkins DJ (2018). Axogenic mechanism enhances retinal ganglion cell excitability during early progression in glaucoma. Proc. Natl. Acad. Sci. USA 115, E2393–E2402. 29463759 10.1073/pnas.1714888115 PMC5877940

[R22] Sanes JR, and Lichtman JW (1999). Development of the vertebrate neuromuscular junction. Annu. Rev. Neurosci. 22, 389–442. 10202544 10.1146/annurev.neuro.22.1.389

[R23] Schwartz GW, Okawa H, Dunn FA, Morgan JL, Kerschensteiner D, Wong RO, and Rieke F (2012). The spatial structure of a nonlinear receptive field. Nat. Neurosci. 15, 1572–1580. 23001060 10.1038/nn.3225 PMC3517818

[R24] Shen N, Wang B, Soto F, and Kerschensteiner D (2020). Homeostatic Plasticity Shapes the Retinal Response to Photoreceptor Degeneration. Curr. Biol. 30,1916–1926.e3. 32243858 10.1016/j.cub.2020.03.033 PMC7239754

[R25] Tien N-W, Soto F, and Kerschensteiner D (2017). Homeostatic Plasticity Shapes Cell-Type-Specific Wiring in the Retina. Neuron 94, 656–665.e4. 28457596 10.1016/j.neuron.2017.04.016 PMC5477664

[R26] Tran NM, Shekhar K, Whitney IE, Jacobi A, Benhar I, Hong G, Yan W, Adiconis X, Arnold ME, Lee JM, (2019). Single-Cell Profiles of Retinal Ganglion Cells Differing in Resilience to Injury Reveal Neuroprotective Genes. Neuron 104, 1039–1055.e12. 31784286 10.1016/j.neuron.2019.11.006 PMC6923571

[R27] Tsukamoto Y, and Omi N (2013). Functional allocation of synaptic contacts in microcircuits from rods via rod bipolar to AII amacrine cells in the mouse retina. J. Comp. Neurol. 521, 3541–3555. 23749582 10.1002/cne.23370 PMC4265793

[R28] Tsukamoto Y, and Omi N (2017). Classification of Mouse Retinal Bipolar Cells: Type-Specific Connectivity with Special Reference to Rod-Driven AII Amacrine Pathways. Front. Neuroanat. 11, 92. 29114208 10.3389/fnana.2017.00092 PMC5660706

[R29] Wässle H, Puller C, Müller F, and Haverkamp S (2009). Cone contacts, mosaics, and territories of bipolar cells in the mouse retina. J. Neurosci. 29, 106–117. 19129389 10.1523/JNEUROSCI.4442-08.2009 PMC6664901

[R30] Yoshimatsu T, D’Orazi FD, Gamlin CR, Suzuki SC, Suli A, Kimelman D, Raible DW, and Wong RO (2016). Presynaptic partner selection during retinal circuit reassembly varies with timing of neuronal regeneration in vivo. Nat. Commun. 7, 10590. 26838932 10.1038/ncomms10590 PMC4742908

