# Supplementary material for: Disassembly and rewiring of a mature converging excitatory circuit following injury
Source: Cell Rep. Author manuscript; Available in PMC 2021 Aug 23. (PMC8381591; doi:10.1016/j.celrep.2021.109463)
Supplement: 1 [file NIHMS1730370-supplement-1.pdf]

Cell Reports, Volume *36*

Supplemental information

Disassembly and rewiring of a mature converging excitatory circuit following injury

Luca Della Santina, Alfred K. Yu, Scott C. Harris, Manuel Soliño, Tonatiuh Garcia Ruiz, Jesse Most, Yien-Ming Kuo, Felice A. Dunn, and Yvonne Ou

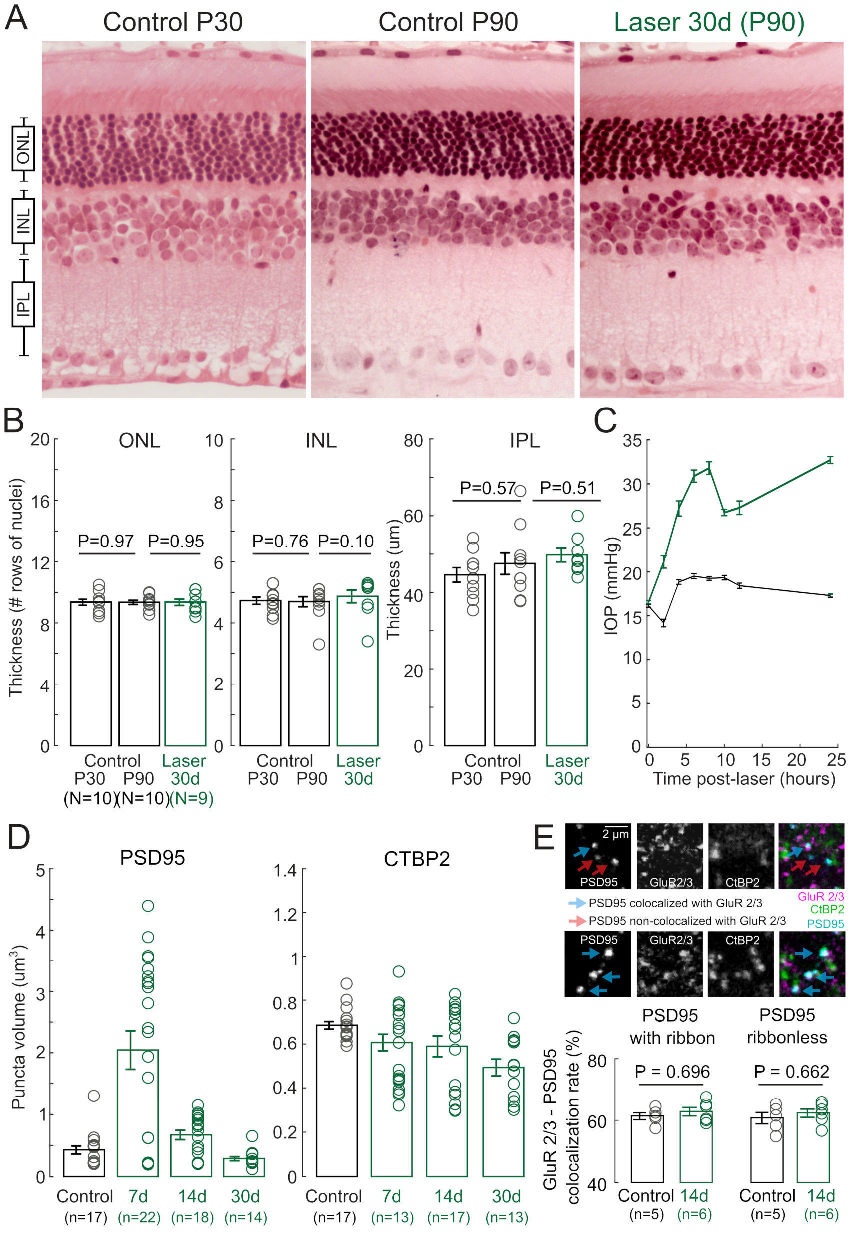

Figure S1. Characterization of the LIOH model, Related to Figure 1

A: Representative vertical sections from control retinas at P30 (left), P90 (middle) and after 30 days from laser (age P90, right) labelled with Hematoxylin-Eosin. ONL (outer nuclear layer, INL (inner nuclear layer) and IPL (inner plexiform layer) limits are delineated next to the sample images.
B: Thickness of nuclear layers (ONL, INL) and synaptic layer (IPL). N = number of animals.
C: Intraocular pressure profile in the first 24 hours following laser induced ocular hypertension. Lasered eyes (green) versus contralateral control eyes (black). N=8 animals.
D: Volume of PSD95 puncta expressed by A_{ON-S} RGCs (left panel) and volume of CtBP2 puncta in the corresponding IPL sublamina. n=number of A_{ON-S} RGCs analyzed from N=13 animals per group.
E: Colocalization rates of ribbon-apposed and ribbonless PSD95 with GluR 2/3 receptors in individual AON-S RGCs from control retinas and after 14 days from laser. n = number of cells from N = 5 animals. Top panels: example of individual PSD95 puncta either colocalized (blue arrow) or non-colocalized (red arrow) with GuR 2/3. Bar plots: mean±SEM. Circles: Individual values. P-values: Rank-sum comparisons between groups indicated by lines.

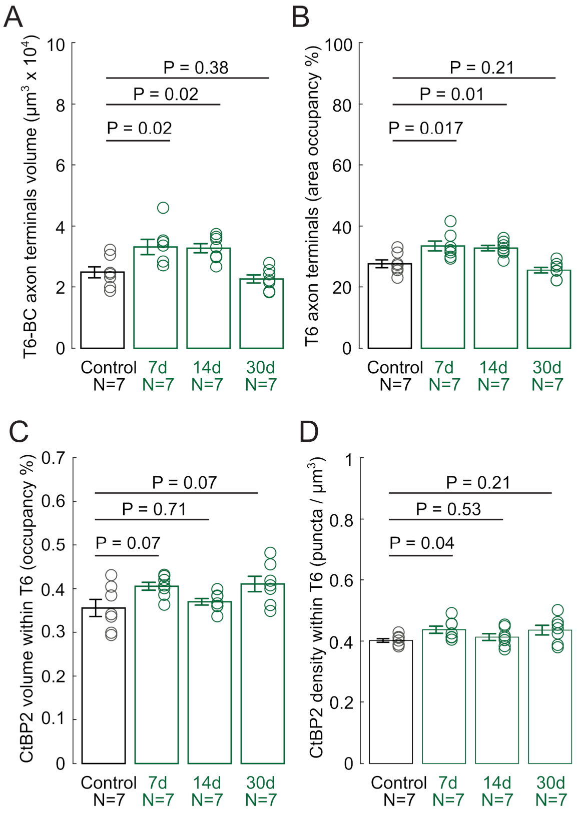

Figure S2. Axon terminals of T6-BCs, Related to Figure 2

A: T6-BC cell axon terminal volume within a 200x200µm IPL region of the whole mount retina. N=number of animals.
B: T6-BC axon terminal area occupancy within a 200x200µm IPL region (projection along IPL depth) of the whole mount retina. N=number of animals.
C: Volume occupancy of CtBP2 within T6-BC axon terminals. N = number of animals.
D: Density of CtBP2 within T6-BC axon terminals. N = number of animals.
Bar plots: mean±SEM. Circles: Individual values. P-values: Rank-sum comparisons between groups indicated by lines.

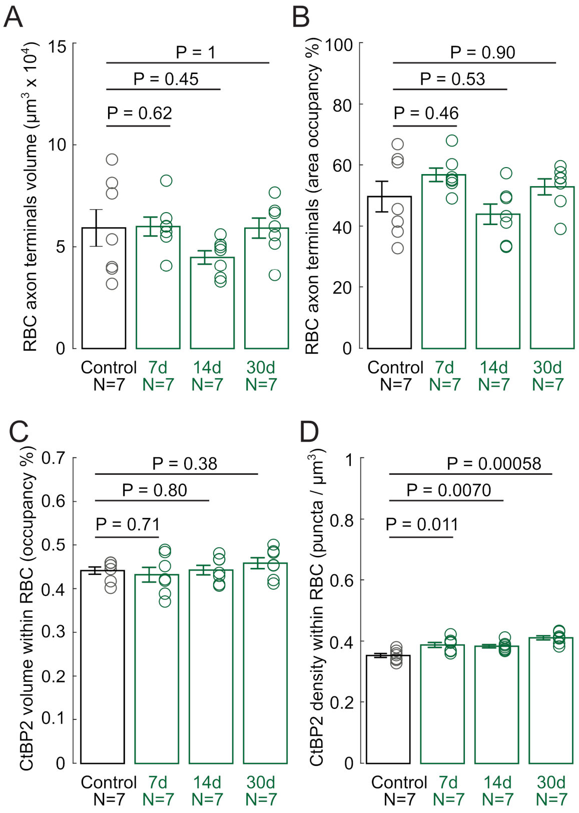

Figure S3. Axon terminals of RBCs, Related to Figure 3

A: RBC axon terminal volume within a 200x200µm IPL region of the whole mount retina. N=number of animals.
B: RBC axon terminal area occupancy within a 200x200µm IPL region (projection along IPL depth) of the whole mount retina. N=number of animals.
C: Volume occupancy of CtBP2 within RBC axon terminals. N=number of animals.
D: Density of CtBP2 within RBC axon terminals. N=number of animals.
Histograms: mean±SEM. Circles: Individual values. P-values: Rank-sum comparisons between groups indicated by lines.

Table S1. PSD95 density on A_{ON-S} RGCs, Related to Figure 1.

Absolute number of PSD95 puncta per A_{ON-S} RGC for each experimental group. Related to Figure 1.

	**PSD95 density per unit-length dendrite (puncta/µm; mean±SEM)**
**Control**	0.507±0.026
**LIOH 7d**	0.264±0.034
**LIOH 14d**	0.288±0.045
**LIOH 30d**	0.373±0.038

Table S2. Intensity-response values for A_{ON-S} RGCs peak currents across rod and cone-mediated light levels, Related to Figure 4.

Light levels and peak response currents measured in whole-cell voltage-clamp recordings of A_{ON-S} RGCs at -60mV to isolate excitatory currents.

**CONTROL**		**LIOH**	
**Rod intensities (Rh*/rod = isomerizations per rod; mean±SEM)**	**Peak current (pA; mean ± sem)**	**Rod intensities (Rh*/rod = isomerizations per rod; mean±SEM)**	**Peak current (pA; mean±SEM)**
0.002±1.9e-05	26.33±6.20	0.002±0	15.56±5.05
0.007±6.39e-04	346.19±166.94	0.007±0.001	67.52±35.28
0.018±1.52e-04	616.25±338.96	0.017±0.001	35.97±12.67
0.055±0.005	890.67±265.43	0.061±0.004	151.16±26.32
0.221±0.020	1157.18±289.23	0.19±0.014	249.02±39.87
0.588±0.005	1259.63±431.77	0.514±0.033	301.54±39.30
1.18±0.01	1318.58±428.06	1.91±0.14	372.06±51.68
		5.35±0.11	425.57±97.21
**Cone intensities (P*/cone = isomerizations per S cone ; mean±SEM)**	**Peak current (pA; mean±SEM)**	**Cone intensities (P*/cone = isomerizations per S cone; mean±SEM)**	**Peak current (pA; mean±SEM)**
0.016±0.001	15.66±2.86	0.015±0.002	7.50±1.26
0.065±0.006	63.23±16.76	0.08±0.005	12.42±2.45
0.17±0.002	375.73±73.72	0.18±0.002	20.30±5.64
0.52±0.048	1139.61±155.96	0.53±0.03	71.81±20.69
2.07±0.19	1896.46±212.34	2.12±0.14	242.73±62.91
5.51±0.06	2152.06±347.91	5.46±0.07	357.19±121.33
		17.24±1.26	497.21±102.96
		45.97±0.45	518.42±152.90
